# Improving comprehension of genetic counseling for hereditary breast and ovarian cancer clients with a visual tool

**DOI:** 10.1371/journal.pone.0200559

**Published:** 2018-07-12

**Authors:** Muy-Kheng M. Tea, Yen Y. Tan, Christine Staudigl, Birgit Eibl, Romana Renz, Ella Asseryanis, Andreas Berger, Georg Pfeiler, Christian F. Singer

**Affiliations:** 1 Department of Obstetrics and Gynecology, Comprehensive Cancer Center, Medical University of Vienna, Vienna, Austria; 2 Department of Obstetrics and Gynecology, Hospital of the Sisters of Charity Linz, Linz, Austria; Ohio State University Wexner Medical Center, UNITED STATES

## Abstract

**Objective:**

Genetic counseling and testing can be offered to individuals who are at high risk of carrying a breast cancer (*BRCA*) gene mutation. However, the content of genetic counseling could be difficult to understand due to complex medical information. The aim of this study was to investigate if comprehension can be improved with a new genetic counseling tool (NGCT hereafter; a tool that combines complex medical information with pictures, diagrams and tables) as compared to conventional oral-only genetic counseling (CGC).

**Methods:**

207 clients attended genetic counseling for hereditary breast and ovarian cancer at the Medical University of Vienna between February 2015 and February 2016. Seventy clients participated in this study and were allocated into two groups: the first 36 participants received conventional (oral only) genetic counseling (CGC) and the following 34 participants received genetic counseling using a new genetic counseling tool (NGCT), which combines complex information with pictures, diagrams and tables. After genetic counseling, all consenting participants were invited to complete a questionnaire with seven questions evaluating their comprehension of the medical information provided.

**Results:**

Socio-demographic backgrounds were comparable in both groups. Correct responses were significantly higher in the NGCT group compared to the CGC group (p = 0.012). NGCT also statistically improves correct response of Q1 (p = 0.03) and Q7 (p = 0.004).

**Conclusion:**

The NGCT leads to an overall better understanding of the content of a genetic counseling session than CGC alone.

## Background

Breast cancer is the most frequently diagnosed malignancy and the leading cause of death in women [1]. While 90–95% of breast cancer cases occur sporadically, approximately 5–10% are hereditary, with breast cancer gene 1 and 2 (*BRCA1* or *BRCA2* hereafter) being the most common [2, 3]. Up to 15% of women diagnosed with invasive breast cancer have at least one first-degree female relative with the disease [3]. Women with a *BRCA1* or *BRCA2* mutation have an increased risk of not only breast and ovarian cancers but also pancreatic cancers and melanoma. Men with a *BRCA2* mutation have elevated risks for prostate cancer (up to 39%) and breast cancer (up to 10%) [4].

After two decades since the discovery of the *BRCA1* and *BRCA2* genes, genetic testing for hereditary breast and ovarian cancer (HBOC) has become a standard practice for breast cancer patients. Currently, there are several oncologic societies proposing guidelines for the identification of high-risk patients for HBOC based on age at diagnosis and/or the number of breast/ ovarian cancer cases in the family[5–8] [9]. In Austria, similar clinical criteria are used [10, 11]. When an individual is identified to be at high risk for HBOC, the individual will then receive genetic counseling and proper risk assessment before genetic testing. As such, genetic counseling and risk assessment are an important part of the process for diagnosis for HBOC patients. It is necessary that patients understand the implications of genetic testing so they can make an informed decision regarding disease management and prevention strategies. However, medical information provided during genetic counseling is often complex.

An older study reported that information during counseling sessions (or the education materials used) is difficult to understand by patients, especially by those of lower educational background or with poor ‘health literacy’ [12]. Adults with lower health literacy were more likely to report avoiding doctor's visits, to have more fatalistic attitudes toward cancer, to be less accurate in identifying the purpose of cancer screening tests, and more likely to avoid information about diseases they did not have [13]. Since the outcome of genetic counseling could guide clinical decisions and disease management, it is necessary that individuals understand the information presented during consultation, so they can make an informed decision. Thus, the overall aim of this study was to examine if patient comprehension can be improved with a new genetic counseling tool (NGCT hereafter; a tool that combines complex medical information with pictures, diagrams and tables) as compared to conventional oral-only genetic counseling.

## Patients and methods

Between February 2015 and February 2016, a total of 207 individuals were referred to the Department of Obstetrics and Gynecology at Medical University of Vienna/ Vienna General Hospital for genetic counseling and risk assessment for HBOC. The General Hospital is a tertiary hospital and in our center, genetic counseling and risk assessment are provided by certified gynecologists with training in genetic counseling and/or certified psychologists specializing in psycho-oncology with at least five years of training in both clinical psychology and genetic counseling [14]. When an individual presents himself or herself to our center for counseling, general information about HBOC (such as inheritance, risks associated with a mutation, possible testing outcomes and implications of the test results) is usually delivered conventionally to clients without any visual aids. At the end of the session, a detailed personal/family history of cancer is collected, and genetic testing may be offered if he/she fulfils the clinical criteria. If the individual would like to proceed with testing, an informed consent was collected along with a blood sample for molecular analysis.

Of 207 individuals who presented themselves to our center for the first time, 75 agreed to participate in the study. All participants were above 18 years of age. All participants were allocated into two different groups: the first 38 participants received conventional oral genetic counseling (CGC) and the following 37 participants received genetic counseling using the NGCT. The new tool was developed for HBOC clients by a panel of certified clinicians and psycho-oncologists based on their experience and feedback from clients during counseling sessions. General information about HBOC is provided in the tool using language suitable for layperson and with pictures, diagrams and tables. Once the tool was developed, the panel validated the content internally within the genetics team in the department; those who were involved in the process are named authors in this manuscript. They were asked to comment on overall relevance, possible usability, number of items, response alternatives, wording, items to delete, missing items, or any additional comments. Disagreements were discussed, and a consensus was achieved through a third person (RR), who is the psychologist in the team and is knowledgeable in lay communication. A final edit of the tool was performed thereafter. The tool then serves as a guide for patients during counseling session (see S1 File; original version in German). Five clinicians and one psychologist involved in providing counseling using the new tool received prior training and were instructed to provide verbal information that are consistent with the written material. At the end of the counseling session, all participants who provided informed consent for the study were invited to complete a questionnaire (developed by the same panel of clinicians and psycho-oncologists in our center); see S2 File, original questionnaire in German). The questionnaire included seven multiple-choice questions evaluating their understanding of the medical information provided during counseling as well as their socio-demographic data: age, nationality, native language, religious affiliation, marital status, number of children, level of education, employment status. The answers provided by the two groups were then analyzed using SPSS package (Version 23, SPSS Inc., Chicago, IL, USA). Comparisons of responses for all seven questions between two groups were made using Pearson´s chi-square test or Fisher´s exact test (when sample size is smaller than 5). Descriptive statistics were provided for sociodemographic data, and comparison between groups were also made using Pearson’s chi-square or Fisher’s exact test. Age was provided as mean and comparison between groups was performed using independent samples t-test. The level of significance was p<0.05. This single institution pilot study was approved by the Ethics Commission of the Medical University of Vienna (approval number: 2029/2014).

## Results

Of 75 clients who agreed to participate in the study, only 70 were included in the analysis. Five participants were excluded from statistical analysis because they did not fill out the questionnaire, leaving 36 participants in the CGC group and 34 participants in the NGCT group. The average age of our study population was 44 years (SD = 13; range 18–75 years). There was only one male participant in the study (1.4%). Socio-demographic distribution in both groups was similar, except for their marital status (Table 1).

**Table 1:** Socio-demographic distribution of clients in both study groups.

	Total N (%)	CGC N (%)	NGCT N (%)	p-value
**Nationality**	70 (100)	36 (100)	34 (100)	0,513
Austria	53 (75,7)	27 (75)	26 (76,5)	
Poland	3 (4,3)	1 (2,8)	2 (5,9)	
Turkey	2 (2,9)	1 (2,8)	1 (2,9)	
Germany	1 (1,4)	0 (0)	1 (2,9)	
Italy	1 (1,4)	1 (2,8)	0 (0)	
Bulgaria	1 (1,4)	0 (0)	1 (2,9)	
Serbia	1 (1,4)	0 (0)	1 (2,9)	
Romania	1 (1,4)	1 (2,8)	0 (0)	
Russia	1 (1,4)	1 (2,8)	0 (0)	
Sweden	1 (1,4)	0 (0)	1 (2,9)	
Croatia	1 (1,4)	1 (2,8)	0 (0)	
Bangladesh	1 (1,4)	0 (0)	1 (2,9)	
Philippines	1 (1,4)	1 (2,8)	0 (0)	
USA	1 (1,4)	1 (2,8)	0 (0)	
not stated	1 (1,4)	1 (2,8)	0 (0)	
**Native language**	70 (100)	36 (100)	34 (100)	0,496
German	50 (71,4)	27 (75)	23 (67,6)	
Polish	4 (5,7)	1 (2,8)	3 (8,8)	
Turkish	2 (2,9)	1 (2,8)	1 (2,9)	
Croatian	2 (2,9)	1 (2,8)	1 (2,9)	
Russian	2 (2,9)	1 (2,8)	1 (2,9)	
Italian	1 (1,4)	1 (2,8)	0 (0)	
English	1 (1,4)	1 (2,8)	0 (0)	
Bulgarian	1 (1,4)	0 (0)	1 (2,9)	
Serbian	1 (1,4)	0 (0)	1 (2,9)	
Swedish	1 (1,4)	0 (0)	1 (2,9)	
Bangladeshi	1 (1,4)	0 (0)	1 (2,9)	
Philippine	1 (1,4)	1 (2,8)	0 (0)	
Kurdish	1 (1,4)	1 (2,8)	0 (0)	
not stated	2 (2,9)	1 (2,8)	1 (2,9)	
**Religious affiliation**	70 (100)	36 (100)	34 (100)	0,548
Roman catholic	39 (55,7)	22 (61,1)	17 (50)	
Orthodox	5 (7,1)	2 (5,6)	3 (8,8)	
Islamic	4 (5,7)	1 (2,8)	3 (8,8)	
Jewish	1 (1,4)	0 (0)	1 (2,9)	
Buddhist	1 (1,4)	0 (0)	1 (2,9)	
without confession	18 (25,7)	10 (27,8)	8 (23,5)	
not stated	2 (2,9)	1 (2,8)	1 (2,9)	
**Marital status**	70 (100)	36 (100)	34 (100)	**0,037**
Married	36 (51,4)	21 (58,3)	15 (44,1)	
In a partnership	17 (24,3)	10 (27,8)	7 (20,6)	
Single	10 (14,3)	5 (13,9)	5 (14,7)	
Divorced/separated	7 (10,0)	0 (0)	7 (20,6)	
**Number of children**	70 (100)	36 (100)	34 (100)	0,18
0	24 (34,3)	16 (44,4)	8 (23,5)	
1	17 (24,3)	7 (19,4)	10 (29,4)	
2	19 (27,1)	9 (25,0)	10 (29,4)	
3	7 (10,0)	3 (8,3)	4 (11,8)	
4	2 (2,9)	0 (0)	2 (5,9)	
5	1 (1,4)	1 (2,8)	0 (0)	
**Level of education**	70 (100)	36 (100)	34 (100)	0,608
College/university/ university related academy	27 (38,6)	12 (33,3)	15 (44,1)	
Vocational school/ high school/ grammar school	24 (34,3)	14 (38,9)	10 (29,4)	
(Not completed) compulsory school/ apprenticeship	19 (27,1)	10 (27,8)	9 (26,5)	
**Employment status**	70 (100)	36 (100)	34 (100)	0,321
Employee/officer	40 (57,1)	23 (63,9)	17 (50)	
Pension	12 (17,1)	7 (19,4)	5 (14,7)	
Unemployed	11 (15,7)	4 (11,1)	7 (20,6)	
Maternity leave	3 (4,3)	0 (0)	3 (8,8)	
Student	2 (2,9)	1 (2,8)	1 (2,9)	
Self-employed	1 (1,4)	0 (0)	1 (2,9)	
Not stated	1 (1,4)	1 (2,8)	0 (0)	

The questionnaire consists of seven questions. A total of 21 participants (30%) provided correct answers to all questions. As expected, the total number of participants with all correct responses in the NGCT group was significantly higher than in the CGC group (Χ^2^ = 6.275; p-value = 0.012). Of the remaining 49 participants, 25 (35.7%) had one wrong answer, 14 (20%) had two wrong answers, seven (10%) had three wrong answers and one participant each (1.4%) had 4, 6 and 7 wrong answers. The average number of wrong responses was 1.3 (SD = 1.36). The average number of wrong responses in the CGC group was slightly higher than the NGCT group (1.56 (SD = 1.48) vs 1.03 (SD = 1.17), respectively) but the difference was not statistically significant (p = 0.102).

Table 2 shows the questions and frequency of correct/ incorrect responses from participants. The most frequent incorrect response was regarding ovarian cancer risk (Q3), with 12 (33.4%) participants in the CGC group and 14 (41.3%) in the NGCT group (p = 0.497). The second most frequent incorrect response was about the possible results of genetic testing (Q6), with 12 (33.4%) in the CGC group and 7 (20.6%) in the NGCT group. Unlike Q3, the NGCT group did better for Q6 than the CGC group but it is not significantly different (p = 0.231). While the CGC group did better overall for Q3 and Q5 as compared to the NGCT group, this is not approaching statistical significance.

**Table 2:** How often was each question answered correctly or incorrectly?.

	Total N (%)	CGC N (%)	NGCT N (%)	p-value
** **	**70 (100)**	**36 (100)**	**34 (100)**	** **
**Q1—What does *BRCA1/2* mean?**				**0,03**
Correct answer	62 (88,6)	29 (80,6)	33 (97,1)	
Wrong answer	8 (11,4)	7 (19,4)	1 (2,9)	
**Q2—How high is the risk of BC when there is a mutation in the *BRCA* gene?**				0,599
Correct answer	58 (82,9)	29 (80,6)	29 (85,3)	
Wrong answer	12 (17,1)	7 (19,4)	5 (14,7)	
**Q3—What is the risk of ovarian cancer when there is a mutation in the *BRCA* gene?**				0,497
Correct answer	44 (62,9)	24 (66,7)	20 (58,8)	
Wrong answer	26 (37,1)	12 (33,3)	14 (41,2)	
**Q4—How high is the risk of passing on a mutation to the next generation?**				0,506
Correct answer	62 (88,6)	31 (86,1)	31 (91,2)	
Wrong answer	8 (11,4)	5 (13,9)	3 (8,8)	
**Q5—Who can be a carrier of a *BRCA* mutation?**				0,276
Correct answer	66 (94,3)	35 (97,2)	31 (91,2)	
Wrong answer	4 (5,7)	1 (2,8)	3 (8,8)	
**Q6—What are the possible results of genetic testing?**				0,231
Correct answer	51 (72,9)	24 (66,7)	27 (79,4)	
Wrong answer	19 (27,1)	12 (33,3)	7 (20,6)	
**Q7—What is the only option to significantly reduce the risk of BC and OC when there is a *BRCA* mutation?**				**0,004**
Correct answer	62 (88,6)	29 (80,6)	33 (97,1)	
Wrong answer	8 (11,4)	7 (19,4)	1 (2,9)	

## Discussion

Overall, our study shows that NGCT improves overall comprehension of medical information with significantly more individuals getting 100% of the questions correct. Among participants who were presented with the NGCT, 44% managed to answer all the questions correctly in contrast to only 17% in the CGC group (p = 0.012). Compared to other studies where some form of visual guide/decision aid has been used to guide counseling, our study showed similar outcome [15–21]. This is expected since visual representations, such as graphs and pictures, can be used to aid communication and help increase the understanding of medical information among patients [22, 23]. For example, Whelan et al demonstrated that patients with early breast cancer who received medical consultation with a visual aid had better knowledge of the disease and treatment options (and greater satisfaction when making decisions regarding adjuvant chemotherapy) as compared to those who received standard consultation alone[24].

Our study also demonstrates that the NGCT statistically improves correct response of Q1 and Q7. Visual presentation of the answers in the NGCT might have served as a memory trigger for participants. For example, the word “genes” occurring throughout the tool might have highlighted the important message that *BRCA1/2* means breast cancer genes, thus leading to more correct responses for Q1 from the NGCT group compared to those where information was communicated through words alone. As for Q7, the NGCT group might have fared better than the CGC group because the message came across stronger and more memorable from the graph, where risks of both cancers were shown to be reduced dramatically after surgery. Although not statistically significant, the NGCT group also elicited more correct responses than the CGC group for Q2, Q4 and Q6. Of all three questions, the NGCT group scored lowest for Q6. Twenty-seven percent of all participants were not able to recall the three potential results of a gene test (i.e. pathogenic mutation, unclassified variant and no mutation). While the information presented in the table is color-coded in the tool (i.e. black, white and grey depending on risk-association), it did not relay meaningful risk-association (and its implications) to participants. Participants could possibly have benefited with a different graphical presentation or different colors highlighting the degree of risk and consequences associated with type of mutation.

Interestingly, the tool did not improve the responses for Q3 and Q5. The NGCT group did not fare better than the CGC group regarding the risk of ovarian cancer when there is a mutation in the *BRCA* gene (Q3). This might be because participants are generally unaware of the fact that having a *BRCA* mutation could also increase their risk for ovarian cancer. Participants might have been misled by its colloquial name i.e. breast cancer genes, and therefore pay less attention when the issue of ovarian cancer is discussed during counseling. Another reason could also be that the information provided in the tool was not sufficiently clear. We have incorporated breast and ovarian cancer risk on the same page; it might have been clearer for the participants if we had separated ovarian cancer information from that of breast cancer. The NGCT group also did not do better than the CGC group when it comes to understanding that both women and men can be carrier of a *BRCA* mutation (Q5). The information was provided in the tool but perhaps it was not emphasized as much as we thought we had.

In addition, clinical management information is often not sufficiently understood by patients, particularly those who are medically uninformed or lack education [12]. Client’s nervousness could also possibly affect information reception and processing since many are highly stressed and uncomfortable when they set foot in a clinic or hospital setting.

Nonetheless, we see an overall improvement in comprehension of complex medical information using NGCT as compared to CGC. Participants were able to elicit the information better when information was presented in the NGCT. However, it is also likely that the tool may have changed the counseling style of a clinician. Clinicians who may have used words that are not easily understood by their patients before are now providing information alongside the tool using words that are easier to understand and more consistent, which then leads to better understanding among patients. This change, however, was not measured in this study.

There are limitations to our study. Study results are based on one institution with high risk HBOC individuals who are presenting for consultation for the first time. As such, individuals who presented at our clinic are likely more motivated with their health and are likely to be more knowledgeable in the medical information presented prior to counseling thereby resulting in better overall comprehension (in the two groups) and further accentuated by the visual tool. The efficiency of the tool could also vary depending on the consulting clinician. As clinicians are confronted with individuals from various backgrounds daily, some may have adapted their language to complement their patient’s health literacy. However, the clinicians and psycho-oncologists involved in the study received prior training and were instructed to provide verbal information that are consistent with the written material. Study sample size is also small due to pilot study, but our results are important and should be validated in larger multicenter studies with an improved version of the NGCT.

According to a systematic review from Garcia-Retamero and Cokely [25], the best type of visual aid depends on the communication goal. Bar graphs are best used to compare several data points, line graphs are best used to depict trends over time, pie graphs to communicate information about proportions, grids to depict very large numbers, magnifier risk scales to depict very small numbers and icon arrays to communicate treatment risk reduction or risk of side effects. To improve accuracy, numerical information should also be depicted in the visual aids. It is also important to learn about the target group and use the appropriate reading level for the group in the tool. As such, we plan to put these points and our findings into consideration and redesign the NGCT. For example, we will separate the risk information for breast cancer and ovarian cancer into two pages, use different color scheme for communicating risk for the possible testing outcomes, and use equal number of photos of both men and women to emphasize that both gender is equally likely to be a carrier of the *BRCA* mutation. We will also change some wordings in the tool, for example, from “there is a 50% chance of inheriting the altered gene (it is the same for every child) and is regardless of gender” to “both men and women could inherit the *BRCA* gene mutation and the risk of passing on the gene mutation to the next generation is 50%. Once these changes are made, we will revalidate the tool within the genetics team and to ensure generalizability, we will also involve the target audience in the evaluation and dissemination of the tool.

## Conclusion

Our study demonstrates that use of the NGCT results in an overall improved comprehension of complex medical information as compared to CGC. It is essential to further research in this area, especially when the outcome of counseling could aid patients in making informed decision regarding screening and prophylactic surgeries. Health care providers attempting to communicate risk information should be trained to provide the meaning of the intended message to their audience, even when guided with a tool. Meaningful messages can help people determine what their best decision will be, whether they are making decisions about everyday health behaviors (e.g. diet) or serious medical situations (e.g. prophylactic surgeries). The meaningful communication of risk should ultimately promote better life outcomes, but more research in this area is needed.

## Supporting information

S1 File The new genetic counseling tool (NGCT). (PDF)

S2 File Questionnaire. (PDF)

S3 File Raw dataset. (XLSX)
