# Supplementary material for: Improving comprehension of genetic counseling for hereditary breast and ovarian cancer clients with a visual tool
Source: PLoS One. 2018 Jul 12;13(7):e0200559. doi: 10.1371/journal.pone.0200559 (PMC6042777; doi:10.1371/journal.pone.0200559)
Supplement: S1 File — (PDF) [file pone.0200559.s001.pdf]

Counseling Guideline

Genetic counseling for hereditary breast and ovarian cancer

ZENTRUM für
Familiären Brust-
und Eierstockkrebs

Motivation

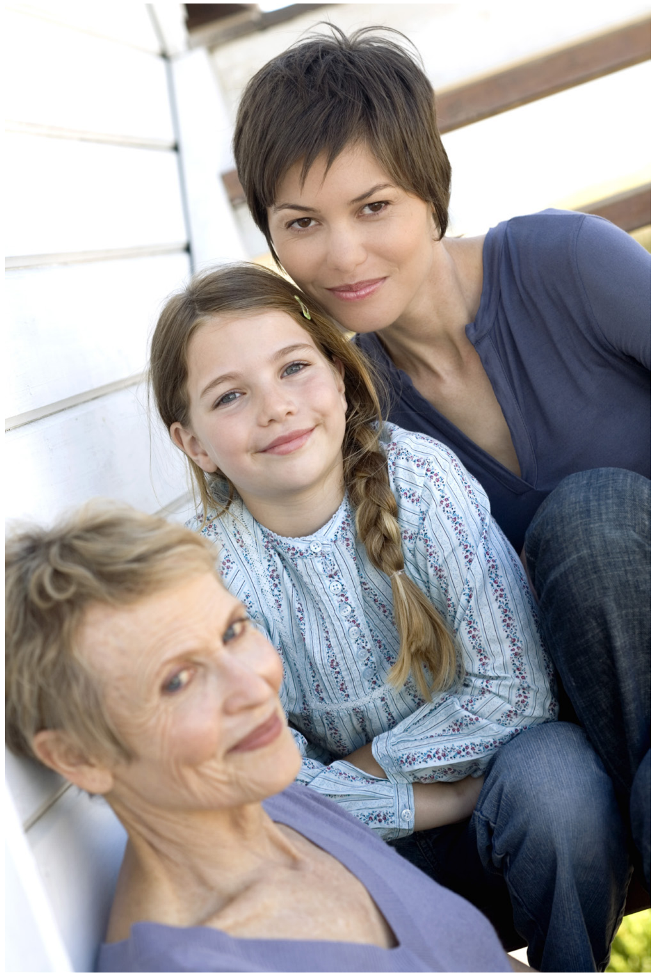

What brings you to genetic counseling today?

ZENTRUM für Familiären Brust- und Eierstockkrebs

Stages of consultation

1. Genetic background
2. Family pedigree creation
3. Decision-making
   - medical indication
   - personal decision
   - possible consequences

Breast and Ovarian Cancer

• One in eight women will develop breast cancer over the course of her lifetime
(lifetime risk: 12.5%)

• One out of every 70 woman will develop ovarian cancer at some point in her lifetime
(lifetime risk: 1-2%)

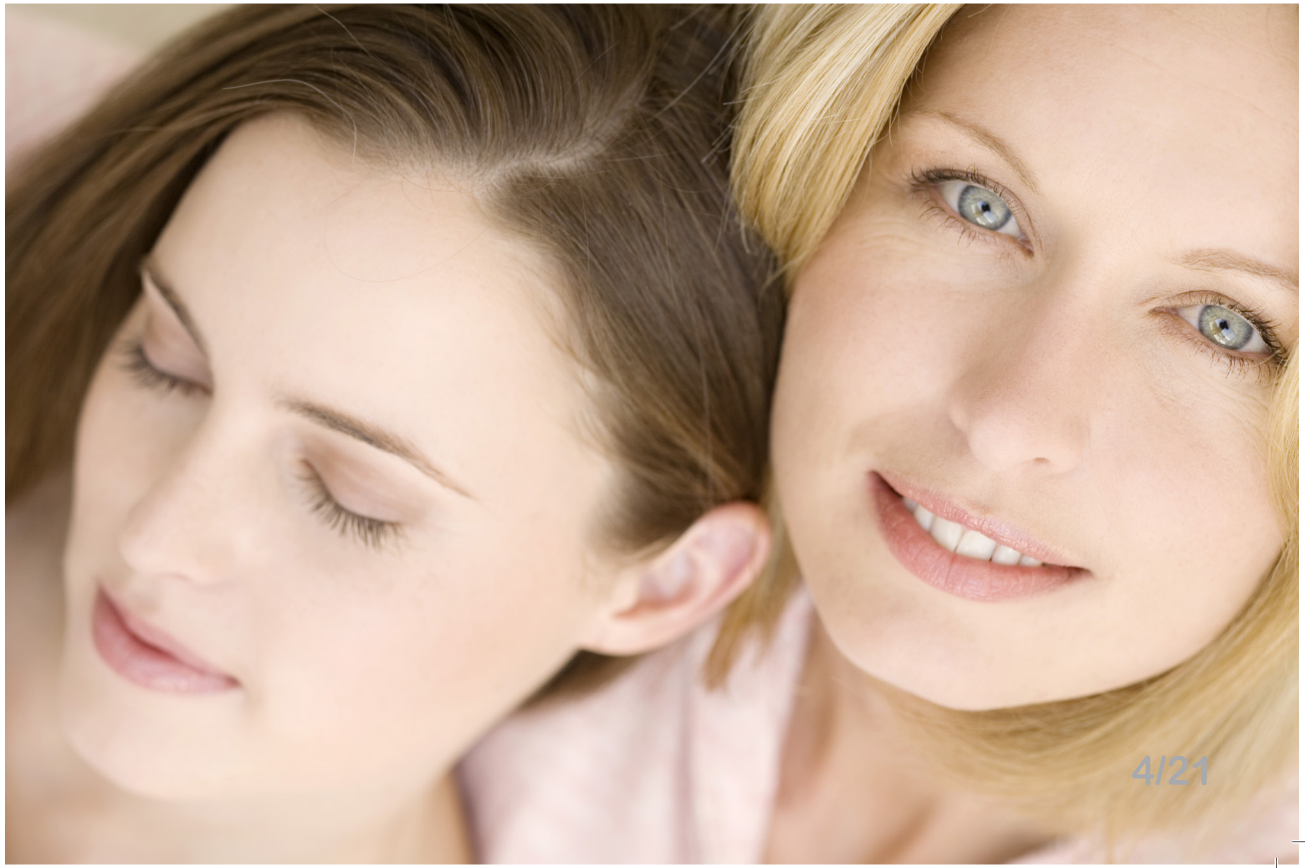

ZENTRUM für
Familiären Brust-
und Eierstockkrebs

Genetic Background

• The complete genetic blueprint for your body is found in the nucleus of each cell in the form of DNA

• DNA = deoxyribonucleic acid (deoxyribonucleic acid)

• Gene: Sections of the DNA that determine features of the human body, e.g. blood type, eye color

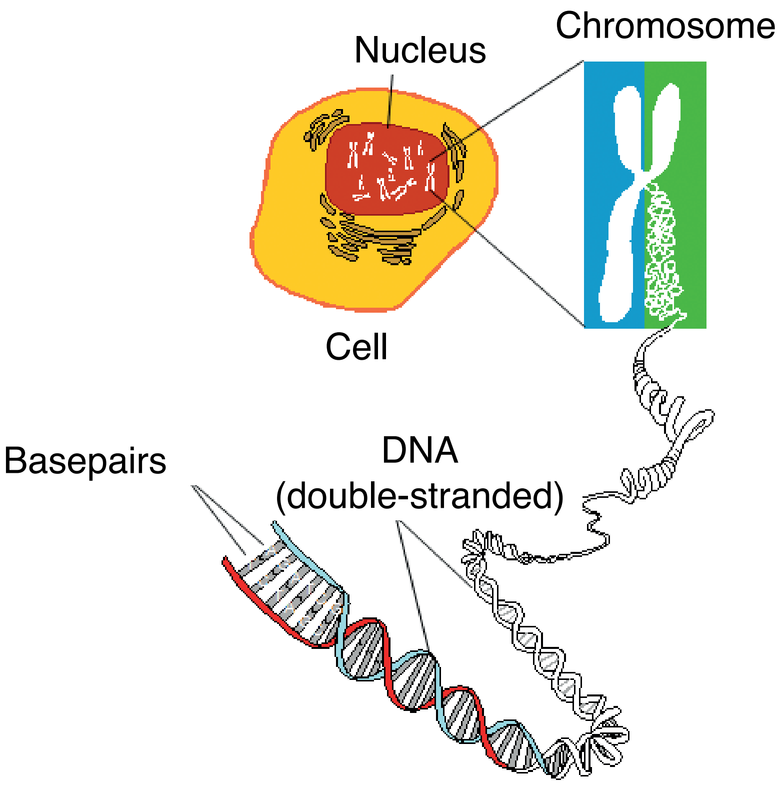

BRCA1 and *BRCA2*

Since 1994 *(BRCA1)* and 1995 *(BRCA2)* were discovered

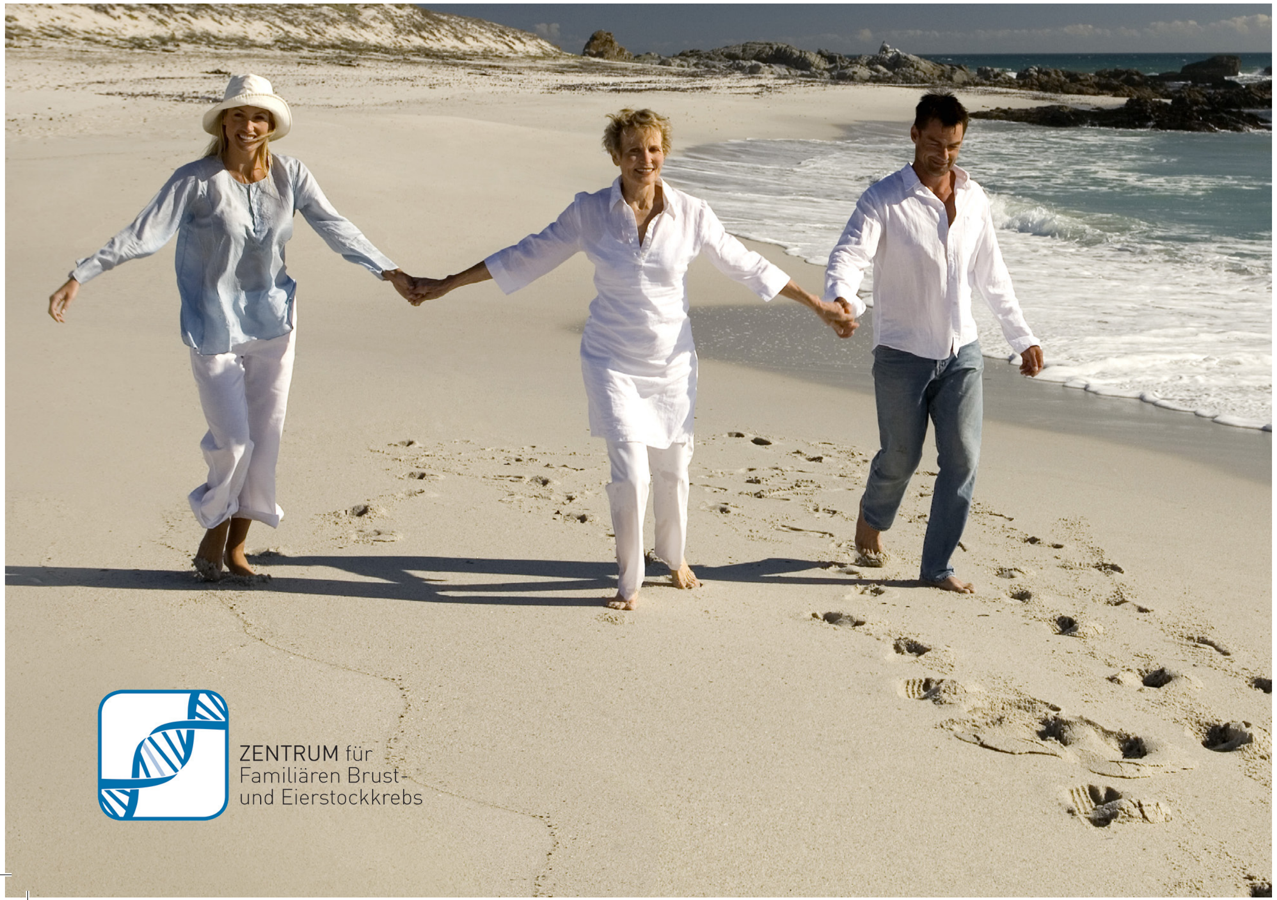

BRCA1 = BReast CAncer 1

BRCA2 = BReast CAncer 2

- *Repair genes*
  (Tumor suppressor genes)

- *They are very important to avoid cancer*

- *Everyone has it (women and men!)*

BRCA1 and *BRCA2*

What happens with a change (= mutation) in one of these genes?

- Gene modification is innate

- Impaired repair function

  - Strong risk increase for breast and ovarian cancer (and low risk of other cancers)

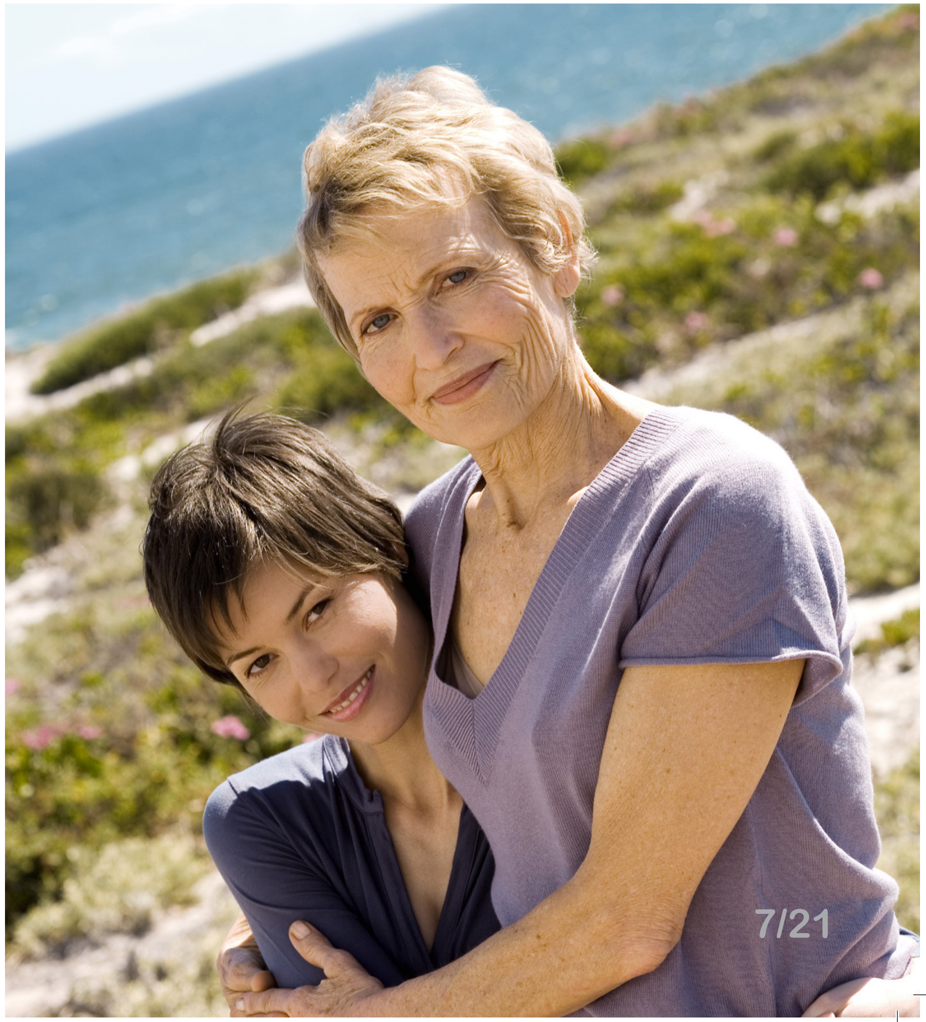

ZENTRUM für
Familiären Brust-
und Eierstockkrebs

Genetic breast cancer

- 90-95% sporadic breast cancer

- 5-10% of all breast cancers are inherited, the causes are genetic changes

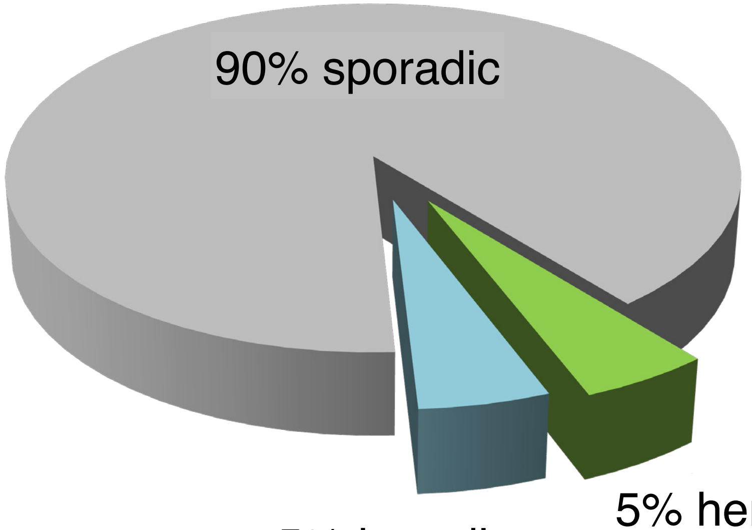

ZENTRUM für Familiären Brust- und Eierstockkrebs

Frequency of BRCA1 / 2 mutations in the population

Rare!

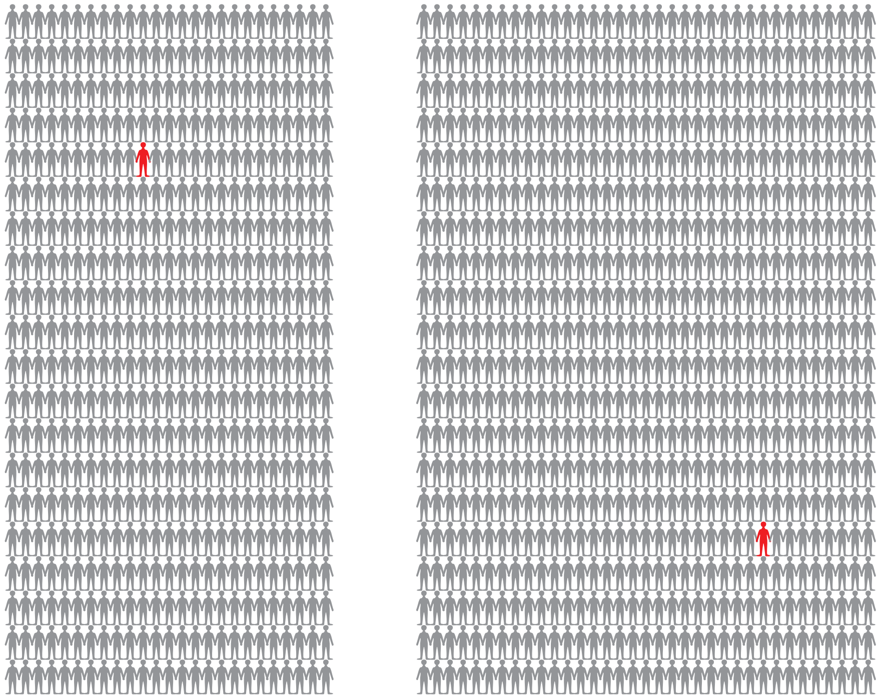

- BRCA1:
1 out of 500 people affected

- BRCA2:
1 of 700 people affected

ZENTRUM für
Familiären Brust-
und Eierstockkrebs

Heredity

- 50% chance of inheriting the altered gene —> it is the same for every child

- Regardless of gender

- Non-carriers cannot pass on genetic change to children
—> the altered gene cannot skip a generation!

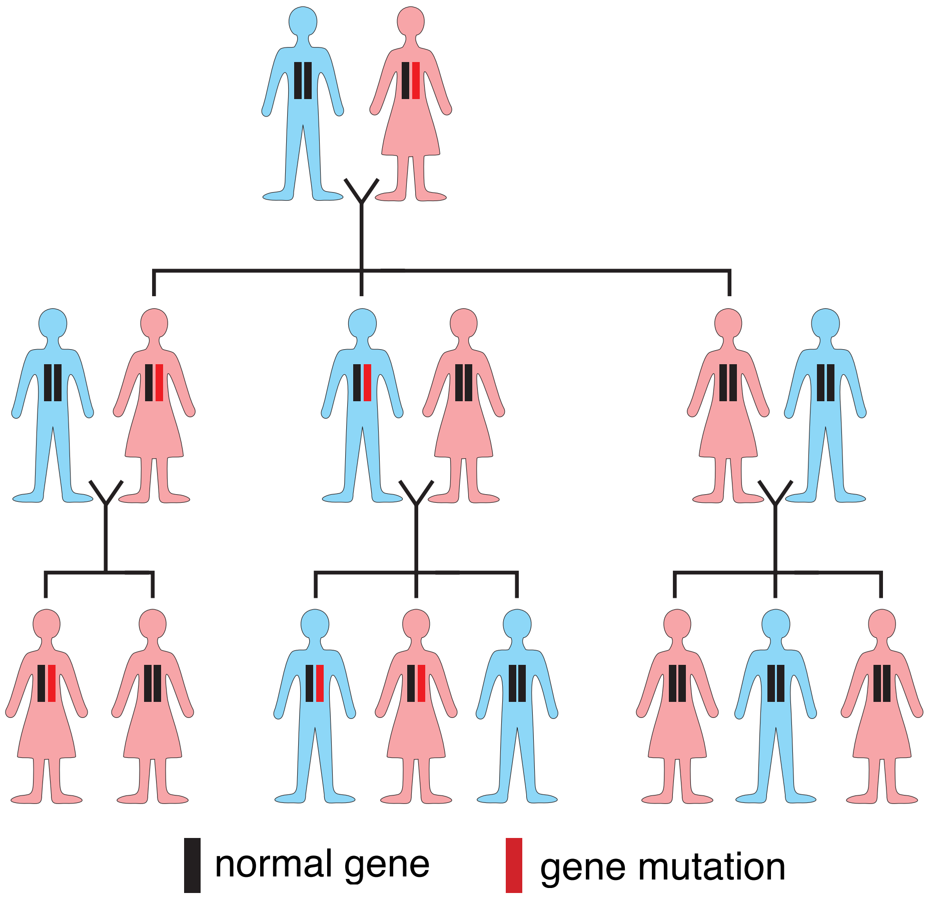

ZENTRUM für
Familiären Brust-
und Eierstockkrebs

Create your family tree

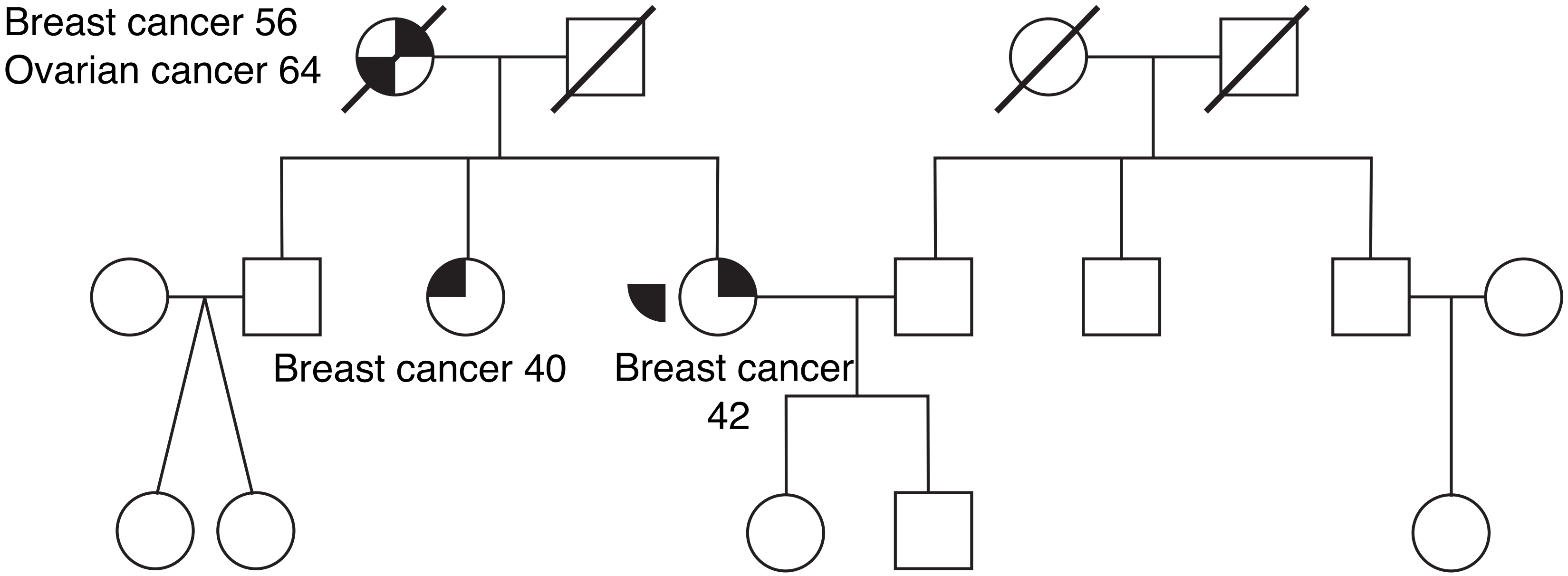

Explanation: Family tree of a sample family. The squares represents male; the circles represents female. Crossed out marks indicate that the person has died. A cancer is marked with a black corner.

ZENTRUM für
Familiären Brust-
und Eierstockkrebs

Medical Indication

• Maternal or paternal line: type and number of cancers, age of onset

• Presence of a familial high-risk constellation?

No

Not high risk;
No genetic testing necessary

Yes

High risk;
Offer genetic testing

ZENTRUM für Familiären Brust- und Eierstockkrebs

Possible results I

(if no genetic mutation was found in the family)

Results	Risk
no mutation	unknown
mutation	**BRCA1: 85% BC, 53% OC**<br>**BRCA2: 84% BC, 27% OC**
unclassified variant	unknown

ZENTRUM für
Familiären Brust-
und Eierstockkrebs

Possible results II

(if genetic mutation was found in the family)

Results	Risk
no mutation	General population
mutation	**BRCA1: 85% BC, 53% OC**<br>**BRCA2: 84% BC, 27% OC**
unclassified variant	unknown

ZENTRUM für
Familiären Brust-
und Eierstockkrebs

BRCA disease risk

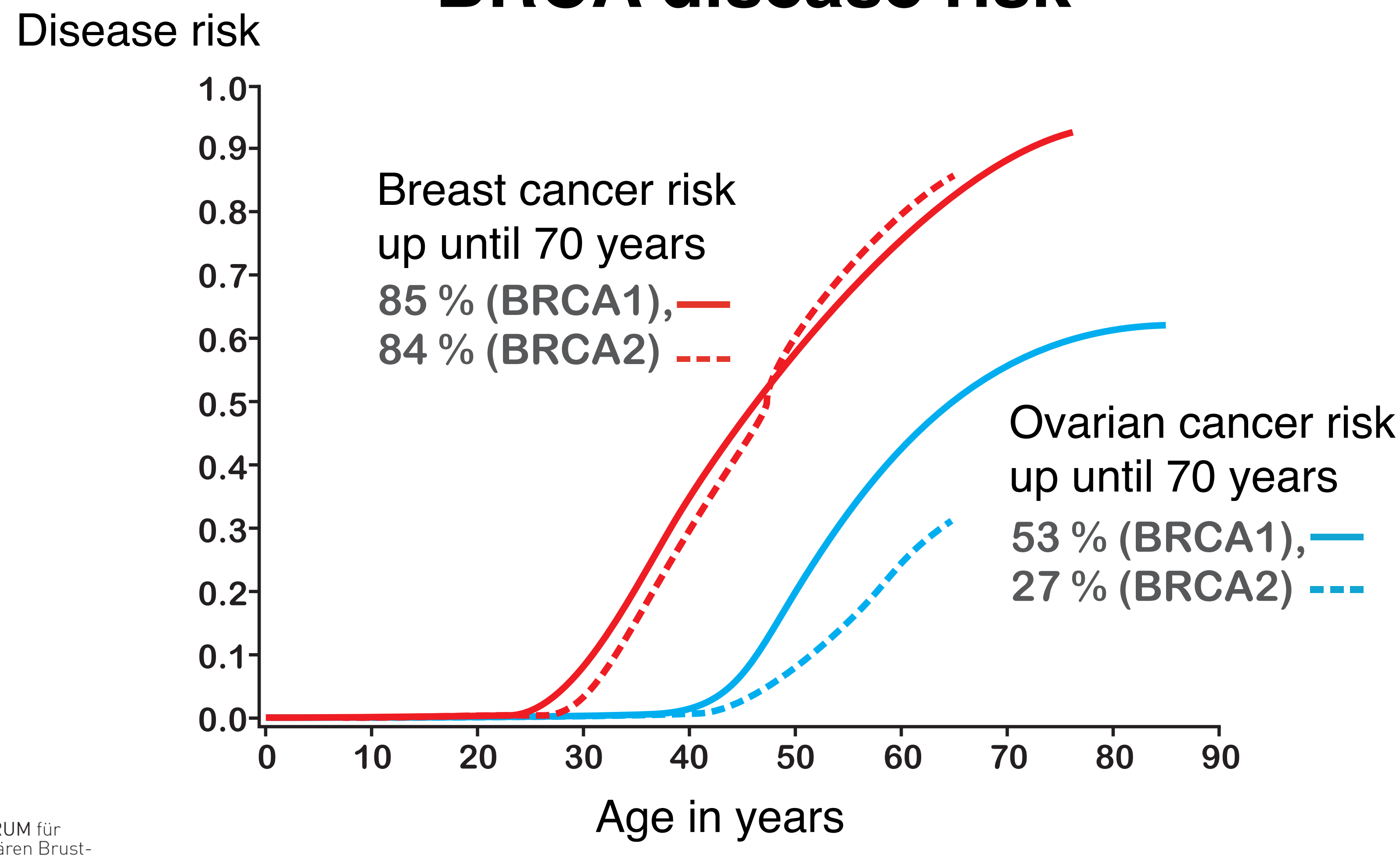

ZENTRUM für
Familiären Brust-
und Eierstockkrebs

Possible medical and psychosocial consequences

Early detection

Prevention

ZENTRUM für
Familiären Brust-
und Eierstockkrebs

Screening program

Type of investigation	From 18y	From 25y	From 35y	if necessary
Gynecological check	Once a year			
Medical breast examination	Once a year			
Breast ultrasound				x
Mammography			Once a year	
Breast MRI		Once a year		
Vaginal ultrasound			Once a year	
Tumor marker			Once a year	

Guideline of the Austrian Society for Senology, Austrian Society for Obgyn., Austrian Society for Surgery, Austrian Society for Radiology

ZENTRUM für
Familiären Brust-
und Eierstockkrebs

Preventive Surgeries

- Preventive removal of the breast tissue (prophylactic mastectomy)

- Preventive removal of ovaries and fallopian tubes (prophylactic ovariectomy, prophylactic bilateral salpingo-oophorectomy)

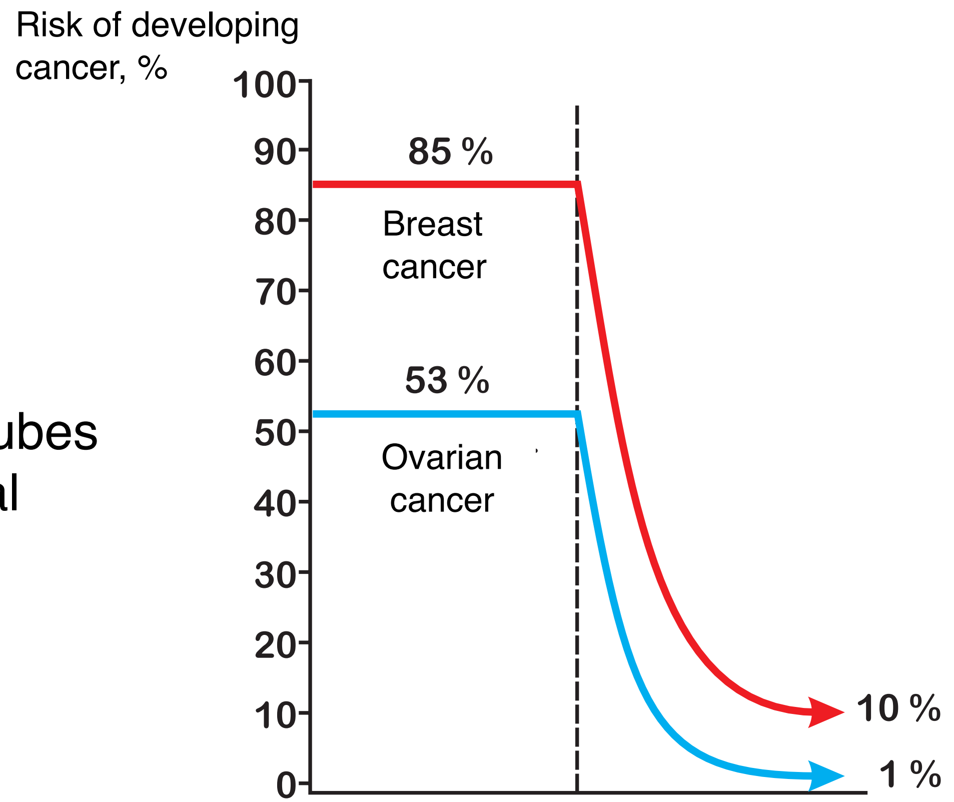

ZENTRUM für Familiären Brust- und Eierstockkrebs

Necessary considerations before an investigation:

• How would you integrate an unfavorable result into your life?

• Which consequences would you presumably draw (e.g. early detection / preventive surgeries etc.)?

• Who would you tell about the gene mutation?

• How would you handle the information that your children might be affected by the hereditary disease risk?

ZENTRUM für
Familiären Brust-
und Eierstockkrebs

Further process

- Invitation to disclosure of test results
- Right to know i.e. to decide if you want to know the result or not

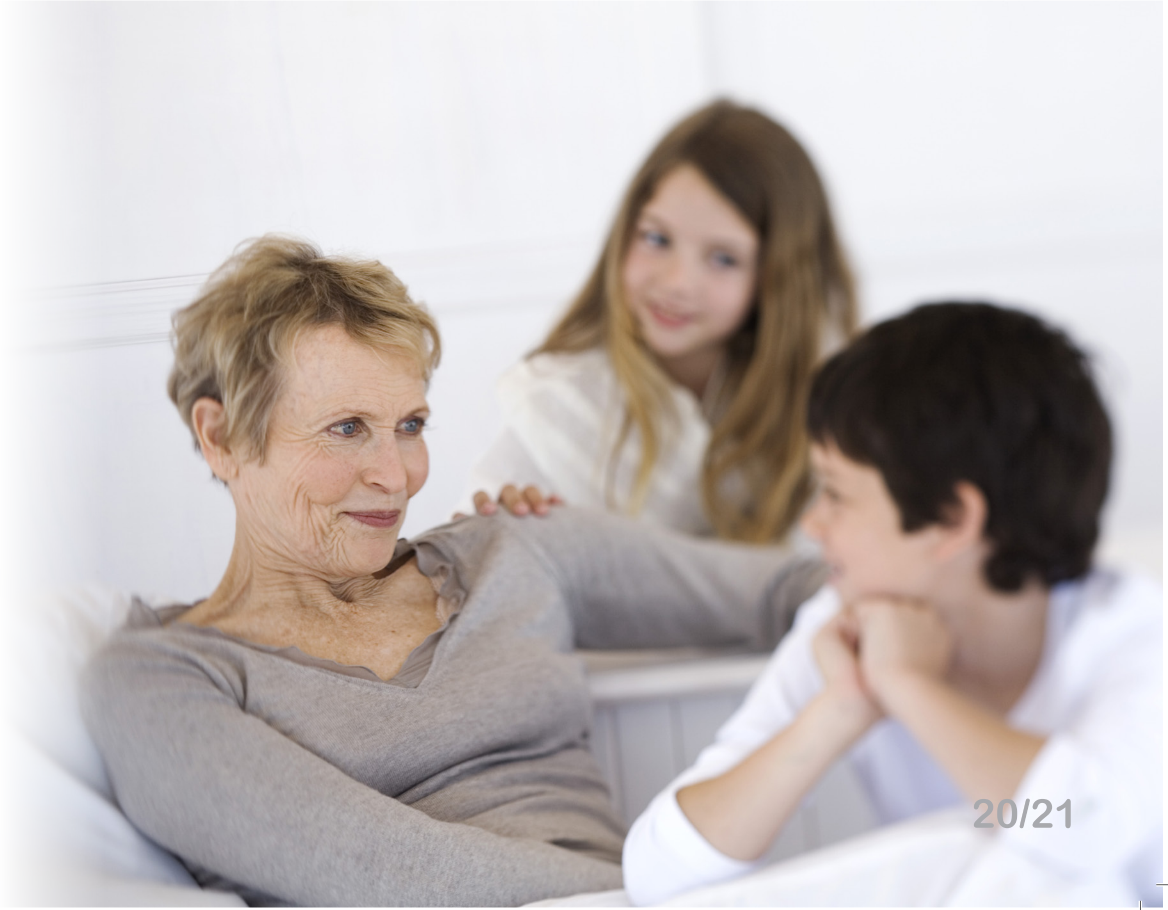

ZENTRUM für
Familiären Brust-
und Eierstockkrebs

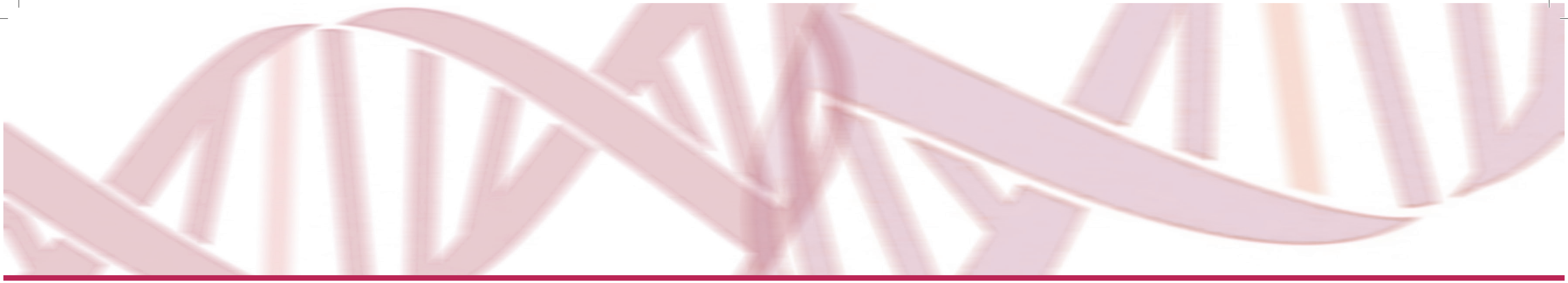

For more information:

www.brustgenberatung.at

ZENTRUM für
Familiären Brust-
und Eierstockkrebs
