# Supplementary material for: Improving comprehension of genetic counseling for hereditary breast and ovarian cancer clients with a visual tool
Source: PLoS One. 2018 Jul 12;13(7):e0200559. doi: 10.1371/journal.pone.0200559 (PMC6042777; doi:10.1371/journal.pone.0200559)
Supplement: S2 File — (PDF) [file pone.0200559.s002.pdf]

S2 File
Questionnaire (with answers in green)

1) What does *BRCA1/2* mean?
A. Breast cancer antibody 1/2
B. Breast cancer gene 1/2
C. A breast-specific hormone
D. A tumor marker

2) How high is the risk of breast cancer when there is a mutation in the *BRCA* gene?
A. 13%
B. 26%
C. 80%
D. 100%

3) What is the risk of ovarian cancer when there is a mutation in the *BRCA* gene?
A. 1-2%
B. 60%
C. 80%
D. No increased risk

4) How high is the risk of passing on a mutation to the next generation?
A. 50%
B. 100%
C. 25%
D. 75%

5) Who can be a carrier of a BRCA mutation?
A. Only women
B. Men only
C. Women after 50 years of age
D. Women and men

6) What are the possible results of genetic testing?
A. Grey result (UV), white result (no mutation), black result (mutation = change)
B. Black result (mutation), white result (no mutation)
C. White result (no mutation), grey result (UV)
D. Black result (mutation), grey Result (UV)

7) What is the only option to significantly reduce the risk of BC and OC when there is a *BRCA* mutation?
A. Once a year mammography
B. Preventive surgery (removal of breast tissue and/or removal of ovaries)
C. Once a year magnetic resonance tomography (MRI)
D. Regular check of tumor markers for breast and ovarian cancer
